# Newly Diagnosed Diabetes Mellitus as an Etiology of Anejaculation and Primary Male Infertility: A Case Report and Review of the Literature

**DOI:** 10.3390/jcm15145718

**Published:** 2026-07-21

**Authors:** Milan Patel, Hannah Moreland, Arya Anvar, Frank Glover, Ankith Maremanda, Nicholas A. Deebel, Joshua Halpern, Robert Brannigan

**Affiliations:** 1 Department of Urology, Loyola University Medical Center, Maywood, 60153 IL, USA; milanp435@gmail.com; 2 Department of Urology, University of South Alabama, Mobile, 36688 AL, USA; 3 Department of Urology, Medical College of Wisconsin, Milwaukee, 53226 WI, USA; 4 Department of Urology, Northwestern University Feinberg School of Medicine, Chicago, 60611 IL, USA; 5 Posterity Health, Centennial, 80112 CO, USA

**Keywords:** azoospermia, diabetes mellitus, male infertility, sexual dysfunction

## Abstract

Diabetes Mellitus (DM) is becoming increasingly prevalent among young men. While DM is known to cause a myriad of health issues ranging from cardiovascular complications to vision loss, its impact on male reproductive health is often unrecognized. In this review, our primary objective is to summarize the literature linking DM and male infertility. We highlight a representative case of a young man who was seen in our clinic for infertility and was ultimately newly diagnosed with DM. Neurovascular changes in diabetic men can gradually lead to the development of sexual dysfunction. DM can also directly impact spermatogenesis through a variety of mechanisms, including the production of oxidative stress and disruption of hormonal pathways. Together, these processes may alter male fertility. When evaluating men with infertility, particularly those with ejaculatory concerns, clinicians must maintain a high index of suspicion for undiagnosed DM. In men with infertility and known DM, improvements in semen parameters are possible with behavioral modifications, weight loss, and improvement in glucose control. Finally, the imminent evaluation and management of a patient with DM and infertility relies on the standard principles of the male reproductive work-up, with emphasis on a few key components as detailed below.

## 1. Introduction

### 1.1. The Impact of Diabetes Mellitus on Men’s Health

In the United States, approximately 15% of adult men have diabetes mellitus (DM)—and about 28% of these men have undiagnosed diabetes [1]. A vast majority of these men have Type 2 DM, as this constitutes 90–95% of total DM cases [1]. Moreover, the prevalence of diabetes mellitus in younger men is rising. Specifically, among U.S. adults aged 20–44 years, the prevalence of diabetes increased substantially between 2009 and 2020 [2]. DM is a chronic disease that carries widespread systemic consequences affecting numerous organ systems [3]. While the cardiovascular and microvascular complications of DM are well understood, the impact of DM on male sexual and reproductive health is less consistently assessed [4,5]. Studies show that men with DM have 3.6-fold higher odds of having erectile dysfunction (ED) when compared to non-diabetic peers [6]. ED also occurs at a younger age and is often more severe in men with DM compared with nondiabetic men [7,8]. DM has also been linked to impaired ejaculatory and orgasmic function; specifically, issues such as retrograde ejaculation, decreased ejaculatory volume, and even anejaculation have been noted [9].

More than one third of men presenting for fertility evaluation do not have an established primary care provider [10]. Thus, given the prevalence of DM among young men and the impact of DM on sexual and reproductive health, the urologist is increasingly at the forefront of care for both DM and fertility. It is imperative for urologists evaluating young men for sexual dysfunction and male infertility to consider the effect of possible undiagnosed DM in their differential. The urologist must both identify the diagnosis, when present, as well as manage the ensuing sexual and reproductive impacts.

### 1.2. Diabetes Mellitus and Male Fertility

Over the course of a male fertility evaluation, issues such as cardiovascular disease and DM are often recognized for the first time. Thus, when diagnoses such as diabetes are suspected, it is critical for the reproductive urologist to encourage the patient to pursue further management with primary care. With the interplay that exists between certain chronic medical conditions and male fertility, a multi-disciplinary approach will optimize the overall medical health of a patient, and thereby, reproductive health. In the case of DM, poorly controlled disease can influence fertility through multiple mechanisms. First, vasculopathy and neuropathy caused by advanced diabetes can impair sexual function [11]. Specifically, diabetic patients may develop erectile and/or ejaculatory dysfunction, which can lead to challenges with intercourse and insemination [9,11]. A growing body of literature has also implicated a more direct link between diabetes and male infertility, citing the negative impact on sperm and semen parameters secondary to factors including oxidative stress, hormonal disruption, and epigenetic changes. Our objective is to present a case report of a patient who was seen in our clinic for reproductive evaluation and eventually diagnosed with diabetes, and to review the literature on the association between diabetes and male infertility.

### 1.3. Case Report:

#### 1.3.1. Patient Presentation

A 28-year-old male with no significant past medical history was referred to our reproductive urology clinic for a chief concern of decreased ejaculate volume. He had been trying to conceive with his female partner for five months, without success. Importantly, the traditional definition of infertility necessitated a period of at least 6–12 months (depending on the age of the female partner) of attempting to conceive a pregnancy without success. However, in 2023, the American Society for Reproductive Medicine (ASRM) released an updated statement which broadened the characterization of infertility with one of the new defining features being “The inability to achieve a successful pregnancy based on a patient’s medical, sexual, and reproductive history, age, physical findings, diagnostic testing, or any combination of these factors” [12].

Thus, our patient and his partner met the criteria for an infertility diagnosis. He had last seen his primary care provider (PCP) about two years prior and over this time period, he noticed a decrease in ejaculate volume; by the time of our initial evaluation, he had developed complete loss of ejaculation but was able to consistently achieve orgasm. He previously had no issues with ejaculatory function. He denied erectile dysfunction. He reported a family history of both type 1 and type 2 DM. His body mass index (BMI) was 24.81 kg/m^2^ (normal range: 18.5–24.9 kg/m^2^). Physical examination was notable for an orthotopic urethral meatus, bilaterally descended testicles (right 18 cc, left 16 cc), bilaterally palpable vas deferens, and normal epididymides. No clinical varicoceles were detected.

#### 1.3.2. Workup

Semen analysis demonstrated aspermia—the complete absence of semen with ejaculation. A post-ejaculate urine (PEU) specimen was obtained, with 87 mL provided in the sample. The sample was concentrated and the pellet was examined; no sperm was found in the specimen. Laboratory evaluation revealed a testosterone of 323 ng/dL, estradiol of 22.2 pg/mL, follicle-stimulating hormone (FSH) of 2.7 mIU/mL, luteinizing hormone (LH) of 10.4 mIU/mL, and prolactin of 4.64 ng/mL. Reference ranges for each respective hormone are located in Table 1. Notably, our threshold of 300 ng/dl as the cut-point for low testosterone is in accordance with the American Urological Association (AUA) guidelines on the management of Testosterone Deficiency [13]. Similarly, we utilize a value of 7.6 mIU/mL as the cut-off value when interpreting FSH, in accordance with the AUA guidelines on the Diagnosis and Treatment of Infertility in Men [14]. The other reference values are based on the ranges suggested by the testing laboratory.

Karyotype analysis was 46XY and Y chromosome microdeletion (YCMD) did not find any deletion in the AZFa, b, or c regions. A hemoglobin A1c (HbA1c) was markedly elevated at >15.5%, with a concomitant random blood glucose of 472 mg/dL. For reference, a normal HbA1c level is below 5.7%, while a value between 5.7–6.4% is characterized as pre-diabetes and a value of 6.5% and above is consistent with diabetes [15]. A random blood glucose level of 200 mg/dL or higher is suggestive of diabetes [15]. Magnetic resonance imaging (MRI) of the prostate demonstrated a 16cc gland with normal contour and without focal lesions, cysts, or evidence of ejaculatory duct obstruction. The seminal vesicles were symmetrical with an anterior–posterior diameter of 8 mm, not concerning for dilation.

#### 1.3.3. Management and Follow-Up

The patient was referred to the emergency department for acute evaluation and urgent endocrinology consultation. He was subsequently diagnosed with Type 2 DM and started on medical therapy. Following initiation of treatment, blood glucose levels improved. On follow up his repeat HbA1c was noted to be 6.8%. He was also initiated on clomiphene citrate for testosterone optimization. A repeat semen analysis with PEU was performed which showed 1 nonmotile sperm. Given lack of sufficient sperm for urine alkalinization with use for Assisted Reproductive Technology (ART), the patient was scheduled for a testicular sperm extraction procedure which is pending at the time of this report.

### 1.4. Pathophysiology of DM-Related Sexual Dysfunction and Infertility

DM adversely affects male sexual function and fertility through a multifactorial interplay of vascular, neurologic, hormonal, and metabolic mechanisms. Chronic hyperglycemia activates several biochemical pathways that culminate in micro- and macrovasculopathy, peripheral and autonomic neuropathy, structural remodeling of penile tissue, and hormonal disturbances, including hypogonadism [11]. Together, these processes can impair both erectile, ejaculatory, and reproductive function.

At the vascular level, diabetes is associated with downregulated expression and activity of endothelial nitric oxide, a critical mediator of penile vasodilation during erection [16]. Hyperglycemia further promotes the formation of advanced glycation end-products, which reduce nitric oxide bioavailability and increase oxidative stress within endothelial cells. In patients with Type 2 DM, accelerated atherosclerosis compounds these effects, leading to reduced perfusion pressure and diminished blood flow to the cavernosal sinusoids, ultimately resulting in erectile dysfunction [11].

Structural changes within the corpora cavernosa further exacerbate erectile impairment. Diabetes is associated with a reduction in elastic fibers and increased collagen deposition within the penile sinusoids, leading to decreased tissue compliance [17]. Heightened oxidative stress and inflammation promote fibrosis and smooth muscle dysfunction, limiting sinusoidal expansion and further compromising cavernosal blood flow.

Neurologic injury represents another central contributor to diabetes-related sexual dysfunction. Diabetic peripheral and autonomic neuropathy lead to diminished genital sensation, delayed or absent orgasm, and impaired coordination of emission and ejaculation [18]. Both somatic and autonomic nerves undergo demyelination and axonal degeneration, disrupting afferent and efferent signaling [9]. Parasympathetic dysfunction impairs activation of the reflexogenic erectile pathway in response to genital stimulation, while compromised pudendal nerve signaling weakens pelvic floor muscle contractions that normally enhance erectile rigidity. Additionally, damage to sympathetic pathways responsible for bladder neck closure and seminal emission may result in retrograde ejaculation or anejaculation [18].

Finally, diabetes-associated hypogonadism contributes to both sexual dysfunction and infertility through disruption of the hypothalamic–pituitary–gonadal (HPG) axis [19]. Reduced testosterone levels and altered gonadotropin signaling negatively affect libido and erectile function. Hypogonadotropic hypogonadism and other broader HPG axis alterations that result from DM can also threaten male fertility more directly, as low testosterone can decrease spermatogenesis [20,21]. Collectively, these mechanisms illustrate how diabetes simultaneously compromises male sexual performance and thereby reproductive potential, underscoring the importance of integrated metabolic and sexual health management in affected patients.

The degree of sexual dysfunction—and its subsequent impact on fertility—may differ depending on the type of DM a patient has. For example, the rate of erectile dysfunction may differ among type 1 and type 2 diabetics. In one study, 26% of men with Type 1 DM reported erectile dysfunction and 37% of men with Type 2 DM reported erectile dysfunction [22]. Similarly, while ejaculatory dysfunction has been linked to patients with both Type 1 and Type 2 DM, it may be more prevalent in patients with Type 2 DM [23]. Lastly, the association between type 2 DM and hypogonadism is well known. However, research into the relationship between Type 1 DM and hypogonadism is more limited, with some data suggesting that individuals with Type 1 DM do not have lower testosterone levels than patients without DM [24,25].

### 1.5. Impact of Diabetes Mellitus on Spermatogenesis and Sperm Quality

DM may predispose to male infertility more directly by its impact on sperm quantity and quality. Multiple studies have investigated changes in semen parameters in men with DM [26,27]. While the collective results have been too inconsistent to draw definitive conclusions, frequently cited semen analysis abnormalities include a reduction in semen volume, sperm motility, and normal morphology [28,29]. DM may exert this potential negative impact on sperm health through a number of mechanisms. First, DM is primarily thought to damage sperm through oxidative stress [30]. High glucose levels, which are often seen in patients with diabetes, increase the production of reactive oxygen species (ROS) [31,32]. ROS play a crucial role in regulating sperm motility, capacitation, and maturation. While a low level of ROS is necessary to promote proper sperm health, the excess level that is commonly seen with DM can lead to oxidative stress [33]. Oxidative stress will result in sperm damage through the accumulation of advanced glycation end products (AGEs), lipid peroxidation, direct injury to nuclear DNA, testicular autophagy, and mitochondrial dysfunction [30,34,35,36,37,38]. Second, hyper-glucosemia may decrease spermatogenesis by disrupting Sertoli cell production and seminiferous tubule organization [39,40]. Third, DM results in an inherent decrease in glucose metabolism and transport, both of which are key to proper sperm motility [41]. Finally, epigenetic changes such as alterations in DNA methylation have been linked with abnormal spermatogenesis, oligospermia, and male infertility [42,43,44,45]. As DM has been broadly associated with epigenetic changes, it has been theorized that this may be yet another mechanism by which DM may lead to infertility [42,43,44,45].

### 1.6. Indications to Consider Diabetes Mellitus Assessment in the New Fertility Patient

When evaluating a man for infertility, the reproductive urologist should assess for DM using a focused history, physical exam, and review of reproductive, sexual, and metabolic indicators. A young patient with otherwise unexplained erectile dysfunction should undergo prompt testing for DM. Similarly, patients of all ages with unexplained ejaculatory dysfunction (i.e., no neurological issues, prior prostate surgery, or use of culprit medications) should undergo testing for DM. In particular, men with low semen volume or a change in semen volume over time should prompt immediate investigation. Other notable “red flag” symptoms include generalized fatigue, polyuria, and polydipsia [46,47,48,49,50]. On exam, central obesity, acanthosis nigricans, and small testicular volume warrant consideration of a diabetes workup [51,52]. As previously mentioned, hormonal abnormalities such as isolated low testosterone or hypogonadotropic hypogonadism can be seen in patients with DM. Finally, we recommend assessing for DM in patients with aspermia or low ejaculate volume. See Table 2 for a summary of the key signs and symptoms.

### 1.7. Evidence for Lifestyle Modifications (Exercise and Diet), Weight Loss, Glycemic Control on Semen Parameters

Lifestyle improvements such as weight loss, improved diet, regular exercise, and glycemic control can influence semen parameters. Obesity is a known risk factor for male infertility and is associated with reduced normal morphology, motility, and count, likely through insulin resistance, chronic inflammation, and oxidative stress [53,54,55]. With respect to blood glucose levels, the available data is limited, but one retrospective study by Wang et al. demonstrated that high-normal fasting blood glucose is associated with impaired semen parameters, including reduced motility as well as an increase in asthenozoospermia rates [56].

Modest weight loss in obese males has been shown to improve sperm concentration and count. Andersen et al. demonstrated a 1.49-fold increase in sperm concentration and a 1.41-fold increase in sperm count in men with obesity who lost on average 16.5 kg body weight on a low-calorie diet [57]. Adherence to healthier diets, including the Mediterranean diet and the DASH (Dietary Approaches to Stop Hypertension) diet, may lead to improvements in sperm count, concentration, and morphology. [58,59,60]. Regular exercise has similarly been linked to improvements in sperm concentration and count, as well as reproductive hormone levels [57,61,62,63]. Lo Giudice et al. demonstrated statistically significant associations between physical activity and sperm concentration, sperm count, motility, and morphology [63]. Together, these data support lifestyle modification as an important factor to consider when managing male infertility.

### 1.8. GLP-1 Receptor Agonists and Related Metabolic Therapies: Implications for Fertility

Glucagon-like peptide-1 (GLP-1) receptor agonists and related incretin-based therapies have been increasingly utilized in the management of type 2 DM and obesity, prompting growing interest in their potential effects on reproductive health. GLP-1 receptors are expressed in multiple organs, including the male and female reproductive tracts as well as the HPG axis, suggesting both central and peripheral mechanisms of action [64]. While many male and female reproductive benefits may be mediated indirectly through significant weight loss, including improvements in female hyperandrogenism, menstrual irregularities, and anovulation, emerging evidence supports potential direct effects of GLP-1 agonists on reproductive tissues [65]. In vitro studies demonstrate that GLP-1 agonists stimulate gonadotropin-releasing hormone secretion, resulting in increased luteinizing hormone release from the pituitary gland. At the gonadal and endometrial levels, these agents exhibit anti-inflammatory and anti-fibrotic properties, with preclinical models demonstrating reversal of polycystic ovary morphology. Similarly, GLP-1 receptors are expressed in Leydig and Sertoli cells, and in vitro data suggest favorable effects on cellular metabolism within the male genital tract [66].

Clinically, GLP-1 agonists have been studied most extensively in women with polycystic ovary syndrome (PCOS), where they have been shown to improve menstrual frequency and metabolic parameters, reduce free testosterone levels, and increase sex hormone–binding globulin concentrations [67,68]. Although fertility-specific outcomes remain incompletely characterized, available studies suggest that GLP-1 agonists may positively influence reproductive outcomes in this population, including both spontaneous conception and assisted reproductive success [69,70].

In men, GLP-1 agonists may mitigate obesity- and Type 2 DM-associated hypotestosteronemia and improve reproductive parameters. Multiple systematic reviews incorporating randomized controlled trials have demonstrated improvements in semen quality—including sperm concentration, total sperm count, motility, and normal morphology—as well as increases in total testosterone levels, particularly among men with underlying metabolic dysfunction [71,72]. Despite these promising findings, robust prospective data evaluating time to conception or long-term fertility outcomes in either sex remain lacking. As GLP-1 agonist use continues to expand among reproductive-age individuals, further investigation is needed to better define their direct versus indirect effects on fertility and reproductive endocrinology.

### 1.9. Reproductive Diagnosis and Management of the Patient with Diabetes

The reproductive evaluation of a male diabetic patient relies primarily on the fundamental principles of the standard male infertility work-up, but with additional emphasis on a few key components. We propose a standard male infertility algorithm to help clinicians navigate the management of diabetic patients. The initial encounter should include a complete male reproductive history and detailed exam, as detailed by the AUA Guidelines [14]. Patients with a known history of DM should be queried in detail on erectile and ejaculatory function, severity of their DM, recent HbA1c level, and adherence to medication management. Patients with erectile dysfunction should be offered one of the numerous therapeutic options including but not limited to phosphodiesterase 5-inhibitors, vacuum erection devices, intracavernosal injections, or penile prosthetic surgery. The approach to ejaculatory dysfunction will be addressed later in this section.

Laboratory assessment is critical for a comprehensive reproductive evaluation in men with DM. At the initial visit, we recommend the clinician order at least one semen analysis. Given the abnormalities in sex hormones that can be seen in men with DM, we also recommend checking levels of the following hormones early on in the evaluation: testosterone, estradiol, FSH, LH [73]. Abnormal hormone levels should be corrected, and underlying disorders may be identified (e.g., Klinefelter syndrome, Kallman syndrome). If a HbA1c level has not been checked within the past year, the urologist may consider ordering this test and contacting the primary care provider for further guidance pending the result. If the clinician suspects poor DM management, the patient should be advised to see their primary care provider to optimize glucose control.

**Figure 1:**
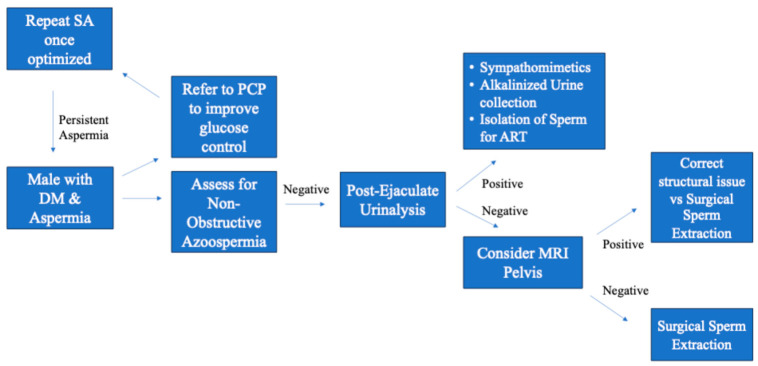
Reproductive Management Algorithm for the Male with Diabetes Mellitus and Aspermia. SA: semen analysis.

After the initial evaluation is complete, most patients can be classified as presenting with normospermia, oligospermia, or azoospermia (obstructive or non-obstructive). Diabetic patients with normospermia, oligospermia or non-obstructive azoospermia should be treated according to the usual principles as outlined by the AUA guidelines [14]. Among diabetic patients with obstructive azoospermia, a subgroup of these patients will have aspermia. See Figure 1 for a summary of the reproductive management algorithm for a patient with DM and aspermia. In patients with DM and aspermia, post-ejaculate urinalysis is indicated. Patients with confirmed retrograde ejaculation may see an improvement in antegrade semen emission with off-label use of medical therapies such as imipramine or pseudoephedrine [74]. Furthermore, sperm from an alkalinized urine specimen can be harvested for assisted reproductive therapy. If sperm is not seen on post-ejaculate urinalysis, we recommend considering an MRI of the pelvis in select patients to rule out structural issues of the reproductive tract such as ejaculatory duct obstruction or congenital abnormalities. Similarly, patients with diabetes who produce an ejaculate, but have reduced volume, should also undergo structural work-up with imaging. If a correctable source of obstruction is identified, either surgical treatment or sperm extraction should be pursued as appropriate; the appropriate decision will depend on other factors including financial implications for the couple as well as the degree of fertility concerns in the female partner. In patients with irreversible or unexplained obstructive azoospermia or non-obstructive azoospermia, we recommend the clinician offer the patient options for surgical sperm extraction.

## 2. Conclusions

While DM is commonplace among the general population, its presence in young men seeking a fertility evaluation is not always immediately recognized. There are numerous pathophysiologic mechanisms in which a DM diagnosis can affect sexual function as well as male fertility. When undiagnosed, this can manifest in extreme presentations such as aspermia due to failure of seminal emission as highlighted in the case report. For many men, a fertility evaluation represents their first touchpoint with the healthcare system in years and allows the reproductive urologist to provide general health counselling and well as offer primary care referral. From a fertility perspective, behavioral modification including diet optimization, weight loss, and exercise is all recommended. Depending on the patient’s initial semen testing, adjunctive testing may be necessary which may influence management for surgical intervention. Future work evaluating the risks and benefits of pharmacologic intervention such as GLP-1agonists is required prior to integration outside of research protocols.

## Figures and Tables

**Table 1:** Reproductive Hormone evaluation.

Hormone	Patient Value	Reference Range	Interpretation
Total Testosterone (ng/dL)	323	300–800	Low Normal
FSH (mIU/mL)	2.7	1.5–7.6	Normal
LH (mIU/mL)	10.4	1.7–8.6	Mild Elevation
Estradiol (pg/mL)	22.2	11.3–43.2	Normal
Prolactin (ng/mL)	4.64	4.04–15.20	Normal

**Table 2:** Signs and Symptoms in the Reproductive Evaluation concerning for DM.

History	Physical Exam	Labs
Generalized fatigue	Central obesity	Isolated low testosterone
Polyuria	Acanthosis nigricans	Hypogonadotropic Hypogonadism
Polydipsia	Small testicular volume	Low Ejaculate Volume
Early onset or unexplained erectile dysfunction		Aspermia
Unexplained ejaculatory dysfunction		

## Data Availability

No new data were created or analyzed in this study. Data sharing is not applicable to this article.
